# Creating a World-Class Program through Training and Certification

**DOI:** 10.3390/bs7040078

**Published:** 2017-11-16

**Authors:** Ann Turner

**Affiliations:** The American Association for Laboratory Animal Science, Memphis, TN 38125-8538, USA; ann.turner@aalas.org

**Keywords:** education, professional development, training, certification

## Abstract

The animal research field has gotten more sophisticated with the use of genetically engineered animals, biohazardous agents, and advanced technologies. Trained and competent personnel are a cornerstone of any animal care and use program. Individual career success is a combination of education, experience, continuing competence, professional development, and personal commitment. Integration of training and certification into programs demonstrate institutional commitment to quality research and enhance the program by providing staff with knowledge and training to address problems and situation that arise and to perform their job in a professional and effective manner. Professional development programs offered through the American Association for Laboratory Animal Science—including the AALAS Learning Library and the AALAS certification process—will be presented.

## 1. Introduction

Those who work with the animals in the laboratory setting have one of the most consequential assignments in the research arena. They provide the foundation for quality research. Professional development can be defined as a combination of education, experience, continuing competence, professional development, and personal commitment. Professional certification is an external recognition of an individual meeting predetermined standards. Professional development programs offered through the American Association for Laboratory Animal Science (AALAS)—including the AALAS Learning Library and the AALAS certification process—serve as examples of professional development resources.

## 2. Delivery Format

There are many ways to deliver training. Traditional classes, which are led by an informed instructor, may be the best method for some types of education, particularly in the area of imparting knowledge to groups. Learning by doing may be best when learning skills and techniques. Some people learn by reading books and articles and others learn better by interacting with a computer or other electronic device, whether individually or in groups. Flexibility and being able to adapt to the learner’s needs is critical to developing an effective training program.

## 3. Key Challenges

Facilities are faced with many challenges in developing a world-class training program. Consideration must be given to who is trained. Varying levels of background, educational level, experience, and understanding are represented in most facilities. Animal care staff requires a wide range of knowledge and skills, encompassing husbandry, animal behavior, observation skills, and a myriad of animal care and research techniques. Research staff require scientific training, as well as research methodology and animal care techniques. Supervisors require advanced training in personnel, budgeting, compliance, management, and human resources. Other audiences which may require training include the Institutional Care and Use Committee (IACUC) or animal ethics committee members who may need training in regulations, decision-making, ethics, and research methodology. While it is not always thought of as training, providing information about your programs, achievements, and progress to institutional officials and decision-makers is critical to organizational success. Consideration should be given to the learning setting because different topics are best delivered by various methods. The objectives of the training must be incorporated in the training planning process and documentation of training is mandatory in many facilities and is desirable even if not mandated. Another challenge is language. While English may be the official language of science, it is often not the native tongue of the people who work in the facility—indeed there may be multiple languages spoken and understood. Each facility must examine the language challenges for their specific site and develop programs to ensure effective communication and work flow.

## 4. Audience Considerations

In developing an institution-wide training program, the goals for four main audiences must be considered: investigator training, both basic and problem focused; animal facility staff training that incorporates new staff; training for more seasoned new members; and lastly training when new species are introduced. Training can also be used as a corrective or remedial action in cases of non-compliance or more frequently for lack of or improper skills. Use of training to improve knowledge or skills is desirable; however, training is not the answer for all problems and should not be viewed as punishment.

The animal research field has gotten more sophisticated with the use of genetically engineered animals, biohazardous agents, and advanced technologies. Trained and competent personnel are a cornerstone of any animal care and use program. Training in laboratory animal science has developed as the field has progressed. Increasingly, technicians are required to be informed of appropriate animal husbandry practices, the characteristics of a variety of laboratory animal species, and the use of modern technology. Because veterinary technicians are widely recognized as valuable personnel for animal research, training programs for veterinary technicians throughout the United States often include laboratory animal science courses in their curriculum.

Institutions have the responsibility of providing animal care and veterinary technicians appropriate training on topics of regulations and ethics of animal research; species-specific biology and husbandry; procedures of animal use; animal welfare in research; facility equipment, operations, and monitoring; and occupational health and safety equipment and practices.

## 5. AALAS

Education and training is primary to the American Association for Laboratory Animal Science (AALAS) as reflected in the Core Values Statement. AALAS programs and services are dedicated to building and disseminating knowledge to those who work in the field of laboratory animal science. AALAS contributes to the professional education of personnel in the laboratory animal field as a way to advance the humane and responsible care and use of laboratory animals. AALAS has over 40 years of experience in the education, certification, and continuing education of laboratory animal technicians and managers. AALAS offers certification programs for technicians and facility managers. The range of educational resources provide by AALAS prepares individuals to work with laboratory animals and supports the professional development of technical and managerial personnel.

The AALAS Technician Certification Program sets professional standards for the advancement of laboratory animal care. The program was developed to recognize professional achievement and provide an endorsement of a technician’s competence in laboratory animal science. AALAS offers technician certification at three levels: Assistant Laboratory Animal Technician (ALAT), Laboratory Animal Technician (LAT), and Laboratory Animal Technologist (LATG). The exams are divided into two principal areas of work-related responsibility: animal husbandry, health and welfare, and facility management. The Certified Manager of Animal Resources (CMAR) program is a combination of demonstrated knowledge of general management and experience and demonstrated knowledge of specific areas of management applied to the laboratory animal facility.

AALAS certification provides employers with confidence that the applicant is competent in the lab animal field. Certification is helpful as a criterion in performance evaluations and promotions. The process of pursuing certification helps the technician to evaluate their strengths and weaknesses, and successful completion of the certification process builds confidence and the knowledge base.

To keep the certification standards current, a job analysis of each level of technician certification is conducted every five years. Based on information gained through the job analysis research, certification resources are updated and the examinations are revised every five years.

In addition to certification resources, AALAS produces a wide variety of general resources. One of the newest programs is webinars. These programs feature experts on diverse topics and are geared for different audiences.

AALAS is perhaps best known for the National Meeting, which is the largest gathering of lab animal professionals in the world. The program features large keynote sessions, lectures, workshops, and seminars. There are over 200 sessions, which allows each attendee to choose the learning format which suits their needs best. An added benefit is ample networking opportunities with people from all over the world. The tradeshow is the largest in the field and features approximately 300 exhibitors.

Another hallmark face-to-face educational opportunity is the Institute for Laboratory Animal Management (ILAM), which is held over a two-year period in Memphis, Tennessee. This intensive program in management lays the foundation for lifetime learning through the ILAM alumni network.

The AALAS Learning Library (ALL) offers a wide range of training options for various audiences in the animal care facility. One suite of courses prepares technicians for the certification examination, while other courses address specific topics in animal husbandry, animal medicine, research methodology and issues, and a vast array of scientific topics. Research staff can meet regulatory and advanced training needs. The ALL also offers courses for IACUC/ethics committee members. These courses help committee members become familiar with what they need to know to fulfill their roles. Species-specific courses are especially helpful for community members or other IACUC members who may not have specific expertise but need to inspect facilities where these animals are housed. The ALL is convenient because it can be accessed anywhere at any time. The ALL can also be used as a training resource to enhance classroom experiences.

Providing world-class education and training for the multiple audiences in the animal laboratory facility is a challenge. Through professional associations, that challenge can be successful.

## Additional Information

https://www.aalas.org/certification

https://www.aalas.org/certification/management-certification

https://www.aalas.org/education/national-meeting

https://aalaslearninglibrary.org/

